# The Intrinsic UV–Visible Fluorescence of Peptides Widely Used for Studying Amyloid Aggregation Devoid of Aromatic Residues

**DOI:** 10.3390/ijms27146453

**Published:** 2026-07-20

**Authors:** Luca Cimmino, Carlo Diaferia, Erika Manicone, Davide Altamura, Elisabetta Rosa, Cinzia Giannini, Luigi Vitagliano, Antonella Accardo

**Affiliations:** 1 IRCCS SYNLAB SDN, Via Ferraris 144, 80146 Naples, Italy; luca.cimmino@synlab.it (L.C.); elisabetta.rosa@synlab.it (E.R.); 2 Department of Pharmacy, Research Centre on Bioactive Peptides (CIRPeB), University of Naples “Federico II”, Via De Amicis 95, 80145 Naples, Italy; carlo.diaferia@unina.it; 3 Institute of Crystallography (IC), National Research Council (CNR), Via G. Amendola 122/O, 70126 Bari, Italy; erika.manicone@uniba.it (E.M.);; 4 Department of Chemistry, University of Bari, Via E. Orabona 4, 70126 Bari, Italy; 5 Institute of Biostructures and Bioimaging, National Research Council (CNR), Via Castellino 111, 80131 Naples, Italy

**Keywords:** non aromatic peptides, self-assembling, photoluminescence, peptide aggregates, bioimaging, diagnostic tools

## Abstract

Non-covalent forces are the primary drivers of biomolecular interactions. They also represent a key factor in the unexpected tendency for proteins, peptides, and even individual amino acids to self-assemble into precise supramolecular assemblies, often characterized by a β-rich, amyloid-like structure. Studies carried out in the last few decades have shown that peptides/proteins self-assembly not only has structural consequences but also generates spectroscopic properties whose origin remains debated. Here, we investigated the spectroscopic properties of four peptides, GAIIGL, NNQQ, SSTSAA, and GNNQQNG (a derivative of the frequently studied GNNQQNY peptide), devoid of aromatic residues, whose crystallographic characterization has been seminal in elucidating the basis of the aggregation process. The spectroscopic behavior of these peptides was analyzed in two different media and in the solid state, highlighting the conditions that favor UV–visible fluorescence emission. These investigations have also prompted the use of this fluorescence as a potential diagnostic tool in nanomedicine. Importantly, the identification of intrinsic fluorescence signatures in these amyloid-like assemblies may support the development of label-free optical approaches for the early detection and monitoring of aggregation-related pathological processes. For all peptides, the emitted fluorescence spans a rather wide range of wavelengths. Although centered in the blue region, a significant signal is also observed in the green region, independent of their physical state. The structural analysis of the solid used to collect the spectroscopic data reveals features related to the packings in the crystal state. This observation suggests that their three-dimensional crystal structures may serve as reliable models for studies aimed at correlating structural and spectroscopic features.

## 1. Introduction

Non-covalent interactions are fundamental to life, determining both the spatial organization of biomolecules and their interactions [1]. For proteins and peptides, the strength of intramolecular non-covalent interactions plays a crucial role in dictating their global flexibility [2,3,4]. Highly structured and rigid scaffolds form when these interactions are strong, whereas they play a limited role in highly flexible biomolecules, such as intrinsically disordered proteins [5,6]. Non-covalent interactions are the driving force behind most biomolecular interactions. They also represent a key factor in the unexpected tendency, highlighted by extensive research in recent decades, for proteins, peptides, and even individual amino acids to self-assemble into precise supramolecular assemblies, often characterized by a β-rich, amyloid-like structure [7,8,9,10]. This propensity has important implications across several seemingly unrelated research areas, ranging from medicine to materials science and engineering [11,12]. Indeed, amyloid-like aggregates are not only formed by these biomolecules involved in the etiology of severe and widespread pathologies, but, being endowed with special mechanical and optoelectronic properties, they are attractive systems in biomaterial sciences [13,14]. In this scenario, the mechanical, biophysical, structural, and spectroscopic characterization of self-assembling proteins and peptides is particularly significant from multiple perspectives.

The seminal studies reported by Eisenberg and coworkers, carried out using peptide models, have provided the first atomic-level information on the aggregation mode(s) of these biomolecules and physical factors that drive self-assembly [15,16]. The importance of these studies remains, even with the availability of more complex, pathology-relevant systems characterized by cryo-electron microscopy [17,18,19], because they offer insights into the structural foundations of peptide aggregation that are relevant to biomaterials science [19]. Notably, the aggregation of peptides and proteins not only creates new biomaterials with interesting mechanical properties but may also produce assemblies that exhibit unexpected intrinsic fluorescence across the visible spectrum (blue, green, and red), often depending on different physical states (solution and solid) [20,21,22,23,24,25,26,27,28]. These investigations have also prompted the use of this fluorescence as a diagnostic tool [29,30]. In particular, the possibility of exploiting intrinsic amyloid fluorescence as a label-free optical readout may open new opportunities for the early detection and monitoring of aggregation-related disorders, overcoming some limitations associated with exogenous fluorescent probes [31,32]. Despite numerous efforts and hypotheses proposed in recent years, the structural and electronic effects underlying this fluorescence, which arise from this specific β-structure motif, remain highly debated [33,34,35,36,37,38]. It cannot be excluded that this multifaceted phenomenon may arise from multiple factors. In this scenario, collecting new spectroscopy data on amyloid-like assemblies and aggregates, especially those that are experimentally characterized, may be crucial for solving this puzzle.

Here, we investigated the spectroscopic properties of peptides whose crystallographic characterization has been seminal in elucidating the basis of the aggregation process [15,16,39,40,41,42,43,44,45,46,47,48], selecting peptides with different polarities and devoid of intrinsically fluorescent residues to better analyze structure-induced fluorescence. In particular, we investigated peptides with the sequences GAIIGL, NNQQ, SSTSAA, and GNNQQNG, a derivative of the frequently studied GNNQQNY peptide, in which the fluorescent tyrosine was replaced with glycine. The aggregation process of all these peptides was biophysically characterized in two distinct media. Structural and morphological insights at the solid state were obtained by Wide-Angle X-ray scattering in Grazing Incidence (GIWAXS) reflection geometry and by Scanning Electron Microscopy. Their spectroscopic characterization, in solution and the solid state, highlighted the specific conditions required for the intrinsic fluorescence emission.

## 2. Results

### 2.1. Selection and Synthesis of the Peptides

For the present study, we chose self-assembling peptides that have been characterized at the atomic level and used extensively as model systems for studying amyloid-like aggregation, but whose spectroscopic properties—especially the intrinsic visible fluorescence—have not been thoroughly investigated [15,16]. In this framework, we selected peptides devoid of fluorescent residues to avoid any interference between this phenomenon and the fluorescence associated with the amyloid-like organization, assuming the characteristic cross-β motif of amyloids in the crystal state. Therefore, we investigated the peptides GAIIGL, a fragment of the human Aβ peptide whose structure is reported in the PDB under code 3PZZ, and SSTSAA, a fragment of bovine ribonuclease A (PDB code 2ONW), which represent two systems with completely different polar and apolar characteristics. In addition, considering the remarkable impact of the prototypal fragment of the yeast protein Sup35 GNNQQNY (PDB codes 1YJP and 2OMM) in structural studies aimed at unraveling the basis of amyloid aggregation, we studied GNNQQNG, a variant composed exclusively of non-aromatic residues, which is predicted to aggregate [49], but has never been experimentally investigated. To further investigate variants of GNNQQNG, we also examined a shorter version with an NNQQ sequence, which has been used as a model system and is characterized by two polymorphs in the crystallized state, where the β-strands are arranged either in parallel or antiparallel fashion (PDB codes 2ONX and 2OLX).

**Figure 1:**
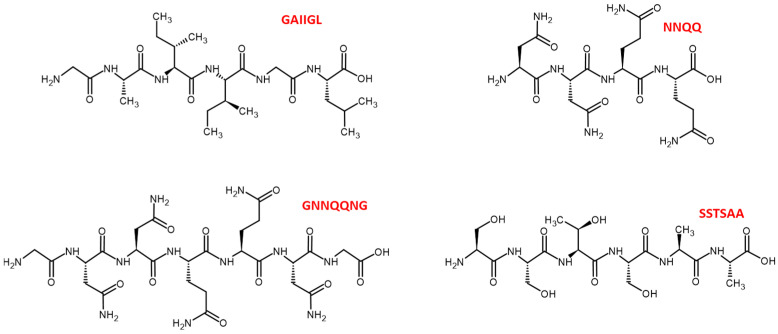
Structural representation of the four model peptides.

All these peptides (Figure 1) were synthesized by SPPS using preloaded Wang resins, which leave a free carboxylic acid at the C-terminus (See Section 4 for details). After the synthesis, the peptides were purified by reverse-phase HPLC, and their identity and purity were confirmed by LC-MS and analytical HPLC on a C18 column (Figures S1–S4).

### 2.2. Studies in DMSO/HEPES

#### 2.2.1. Solution Structural Characterization

**Figure 2:**
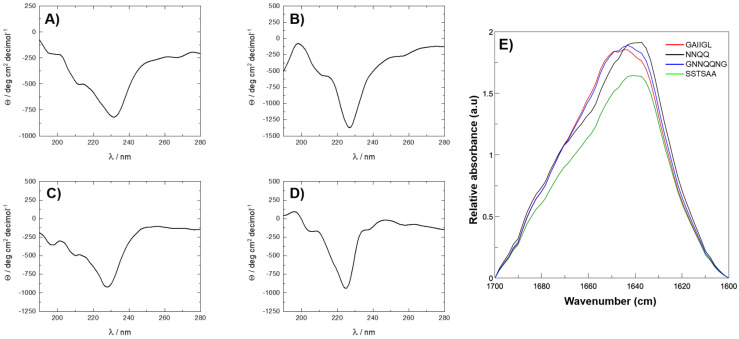
Secondary structure characterization in DMSO/HEPES. Far-UV CD spectra recorded between 280 and 190 nm for: (**A**) GAIIGL, (**B**) NNQQ, (**C**) GNNQQNG and (**D**) SSTSAA at 0.5 mmol/L. (**E**) Deconvoluted FTIR spectra of peptides in the Amide I region, acquired in the same conditions.

The initial characterizations of the peptide aggregation process in solution were performed under conditions emulating those used for their crystallization, as reported by Eisenberg and coworkers [15,16]. After their synthesis, the four lyophilized peptides were first dissolved in DMSO at 5 mM and then diluted to a final concentration of 0.5 mM in 150 mM HEPES buffer (pH 7.4) containing 150 mM NaCl. Immediately after sample preparation, preliminary Circular Dichroism (CD) measurements were collected and were consistent with predominantly disordered (random-coil-like) conformations. Subsequently, the same samples were incubated for 48 h at 37 °C, and new spectra were recorded. As shown in Figure 2A–D, the spectra are dominated by a single negative band in the 225–230 range, with minima at 230, 227, 228, and 225 nm for GAIIGL, NNQQ, GNNQQNG, and SSTSAA, respectively. The presence of this minimum indicates the formation of assemblies, likely amyloidogenic and rich in β-structure.

To gain further insights into the secondary structure assumed by these peptides, FT-IR spectroscopic analyses were performed on dried peptide samples. The deconvoluted spectra in the amide I region (1600–1700 cm^−1^) show that all peptides exhibit a characteristic absorption band centered around 1640 cm^−1^ and a broad signal around 1670 cm^−1^ (Figure 2E). These bands are commonly associated with β-sheet structures, with the latter often assigned to antiparallel β-sheets [50]. However, the absorption around 1670 cm^−1^ may also be influenced by residual trifluoroacetic acid (TFA); therefore, this assignment should be interpreted with caution.

**Figure 3:**
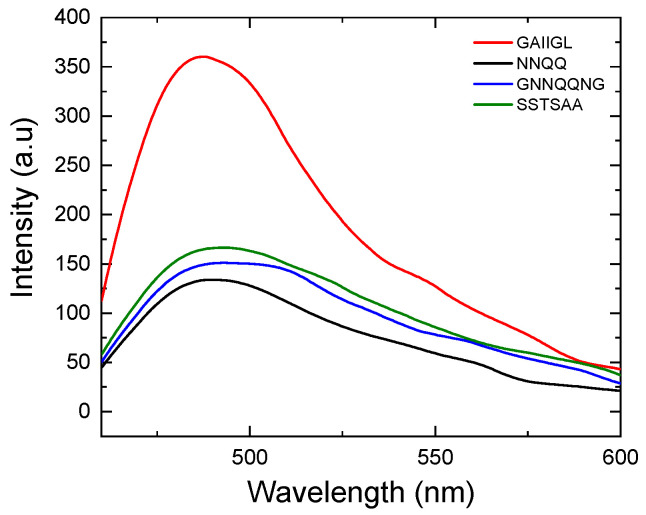
Thioflavin T (ThT) fluorescence emission assay of peptide aggregates formed under the DMSO/HEPES preparation protocol, showing the characteristic ThT emission enhancement upon binding to β-structured amyloid-like assemblies.

The likely amyloid-like nature of these assemblies was further assessed using Thioflavin T (ThT). As shown in Figure 3, the addition of this amyloid probe led to a characteristic ThT emission band with an emission centered in the 480–500 nm region, confirming the formation of amyloids in solution for all peptides.

#### 2.2.2. Solid-State Structural Characterizations of the Aggregates

##### Grazing Incidence Wide-Angle X-Ray Scattering

**Figure 4:**
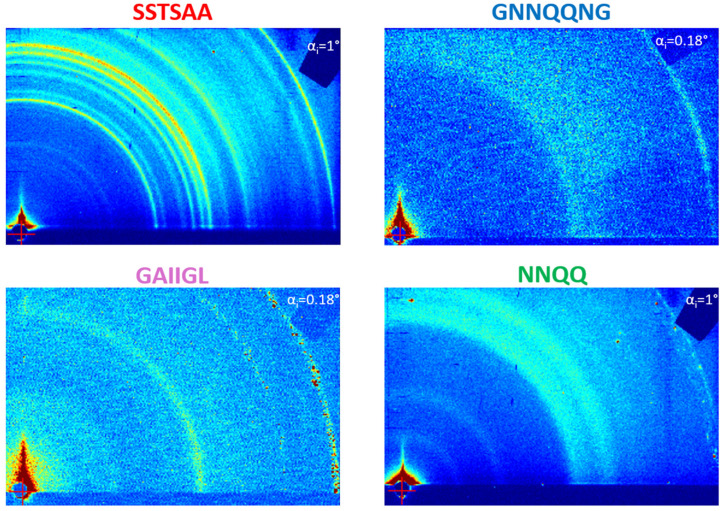
2D GIWAXS patterns collected for the selected peptide sequences. The incidence angle (α_i_) is reported for each measurement.

The tendency of these peptides to form crystalline structures was confirmed by the presence of diffraction rings in the GIWAXS patterns (Figure 4). In the case of the SSTSAA sequence, numerous, much sharper peaks are observed compared to other peptides, indicating a much higher tendency to aggregate into periodic structures with long-range order. Less structured patterns are observed for GAIIGL, NNQQ, and GNNQQNG. No clear preferred orientations were detected, except for GAIIGL, which features a main peak with a slight increase in intensity towards the in-plane direction, also suggesting a higher tendency for anisotropic aggregation. Also, the incidence angle (α_i_) was optimized to maximize the scattering signal for each sample, being α_i_ = 1° for SSTSAA and NNQQ, while α_i_ = 0.18° for GNNQQNG and GAIIGL. Due to the larger incidence angle used in the former case, a larger amount of deposited material is expected for SSTSAA and NNQQ.

1D-folded experimental GIWAXS data (Figure S5) were compared to calculated diffraction patterns expected from amyloid-like assemblies, resulting from the aggregation of β-structure motifs, based on the selected peptide sequences in the original form (GAIIGL, NNQQ, GNNQQNY, SSTSAA), provided by the Protein Data Bank (PDB) as pairs of β-sheets each composed of five strands (Figure S6). An overall qualitative agreement between experimental and calculated data is evident in Figure S5. Although the position and shape of each experimental peak do not faithfully reproduce the calculated profile, a clear correspondence in the whole profile behavior can be recognized, suggesting that crystalline domain anisotropy and dimensions could significantly affect the experimental diffraction profile, although a good match with the hypothesized structural model can be assumed for all the selected peptides. In particular, diffraction peaks can be recognized in GIWAXS patterns relevant to all samples, corresponding to *d*-spacings in the range 4.4–4.8 Å, which can be associated with the cross-β motif of amyloids. Such peaks are highlighted in Table S1 and in the plots of Figure S5 with a superimposed shaded area.

##### Scanning Electron Microscopy Studies

**Figure 5:**
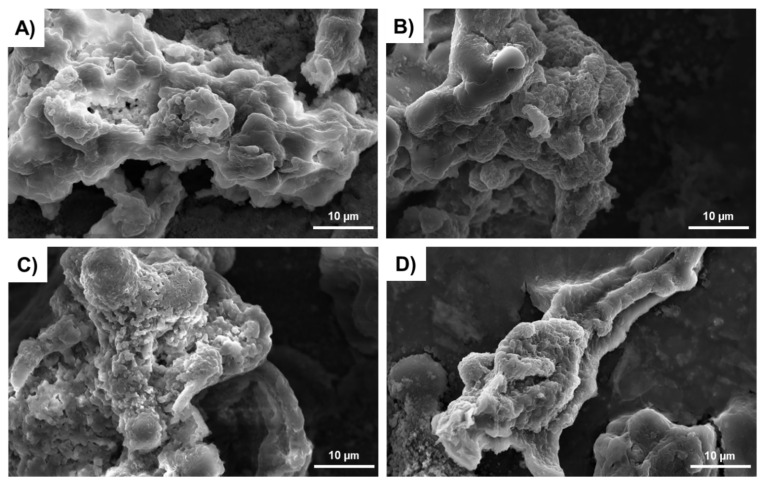
SEM micrographs of supramolecular aggregates formed by the four peptide sequences under the DMSO/HEPES preparation protocol: (**A**) GAIIGL, (**B**) NNQQ, (**C**) GNNQQNG, and (**D**) SSTSAA (scale bars as indicated).

SEM analyses were performed to examine the supramolecular morphology of aggregates prepared using the DMSO/HEPES protocol. At the examined length scale (10 µm), samples showed mostly compact, micrometer-sized agglomerates with rough, folded, or lamellar surfaces (Figure 5). This morphology may be attributed to the coalescence and collapse of supramolecular networks into compact deposits, promoted by drop-casting and drying.

#### 2.2.3. Analysis of the Visible Probe-Free Fluorescence in DMSO/HEPES

**Figure 6:**
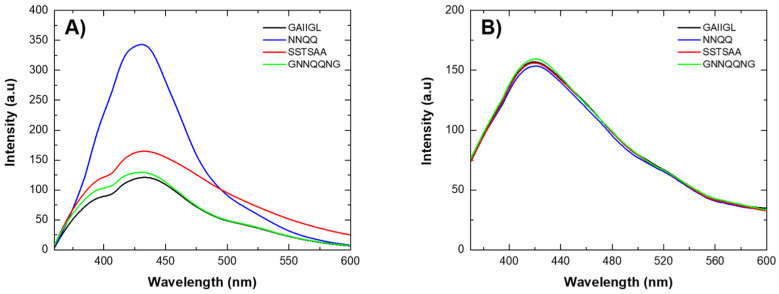
Intrinsic fluorescence spectra of peptide aggregates formed under the DMSO/HEPES preparation protocol, excitation wavelength 350 nm. Fluorescence spectra in: (**A**) solution and (**B**) at the solid state.

The ability of the four peptides to emit a label-free structure-dependent visible fluorescence was investigated both in solution and in the solid state. Solution studies were conducted with peptides dissolved in a 0.5 mM concentration in DMSO/HEPES. In these conditions, all peptides exhibit a broad fluorescence emission in the 400–550 nm interval upon excitation at 350 nm (Figure 6A and Figure S7). The excitation wavelength for the measurements was chosen after preliminary screening between 330 and 430 nm, taking into account the more common wavelengths at which amyloid-like assemblies typically emit fluorescence [20,21,22,23,24,25,26,27,28]. Among the investigated excitation wavelengths, 350 nm yielded the strongest emission, while all the other ones produced either negligible or significantly lower emission intensities. Notably, while the maximum of the emission falls in the near-UV/blue region, a significant signal is also detectable at higher wavelengths (>500 nm, green region).

A rather similar profile emerges from the solid-state analysis. Indeed, the emission collected from the air-dried samples presents a similar dependence on the wavelength, with clear emission both in the blue and green regions (Figure 6B). It may be surprising that the solid samples of the different peptides have distinct 2D GIWAXS patterns (Figure 4) but similar emission spectra (Figure 6B). In this context, it should be noted that the observed fluorescence and X-ray scattering arise from related but distinct structural factors. According to current knowledge (see below), the intrinsic fluorescence of the amyloid-like structures depends on the tight association of peptide chains within the basic element, i.e., the cross-β structure. The observed scattering, which confirms the presence of the main features of the cross-β structure, also depends on the degree of ordering of this element across different peptide samples, i.e., on their crystallinity.

### 2.3. Studies in PBS

In addition to characterizing the peptides in the crystallization solution (DMSO/HEPES), structural and spectroscopic investigations were extended to PBS. Since no a priori information was available on the behavior of the peptides in this medium, we initially performed solubility studies in 10 mM PBS. Given the lack of aromatic residues in these peptides, which precluded reliable UV-based quantification, the experiments involved sequentially adding increasing amounts of lyophilized peptide to a fixed volume of buffer, with gentle mixing. The mixture was then centrifuged (15,000 rpm, 15 min, room temperature) to remove any undissolved material. The apparent solubility was recorded as the highest concentration at which the supernatant stayed clear and free of visible precipitate or turbidity after two consecutive centrifugation cycles under the same conditions. In addition to a general decrease in solubility relative to DMSO/HEPES, these analyses revealed clear differences across the peptide series, with the lowest (1.0 mg/mL; 2 mM) and highest estimated values (16 mg/mL; 30 mM) observed for the hydrophobic GAIIGL and the polar SSTSAA peptides, respectively. Intermediate values were shown by the related peptides NNQQ and GNNQQNG, with the shorter peptide (solubility 10 mg/mL; 20 mM) being more soluble than the longer one (2.0 mg/mL; 3 mM).

#### 2.3.1. Biophysical Characterization

**Figure 7:**
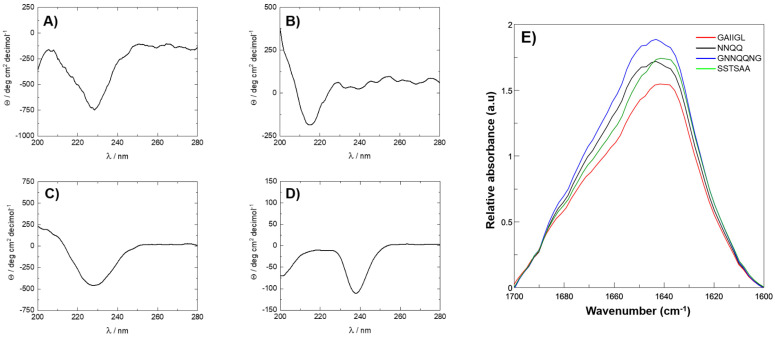
Secondary structure characterization in PBS. Far-UV CD spectra recorded between 280 and 200 nm for: (**A**) GAIIGL, **(B**) NNQQ, (**C**) GNNQQNG and (**D**) SSTSAA at their maximum concentration. (**E**) Deconvoluted FTIR spectra of peptides in the Amide I region.

The biophysical characterization in PBS, performed at the maximum solubility of each peptide, revealed their propensity to form β-rich structures in this medium as well. Indeed, CD spectra show the classical fingerprint of β-sheet formation, being characterized by a single negative minimum (Figure 7A–D). The negative peak is at ~215 nm for NNQQ, whereas it is red-shifted for the other three peptides, in the region 225–230 nm for GAIIGL and GNNQQNG, and ~235–240 nm for SSTSAA. These aggregates were further investigated by FT-IR spectroscopy (Figure 7E) and ThT assay (see Figure S5), which confirmed their amyloid-like structure.

#### 2.3.2. Critical Aggregation Concentration

**Figure 8:**
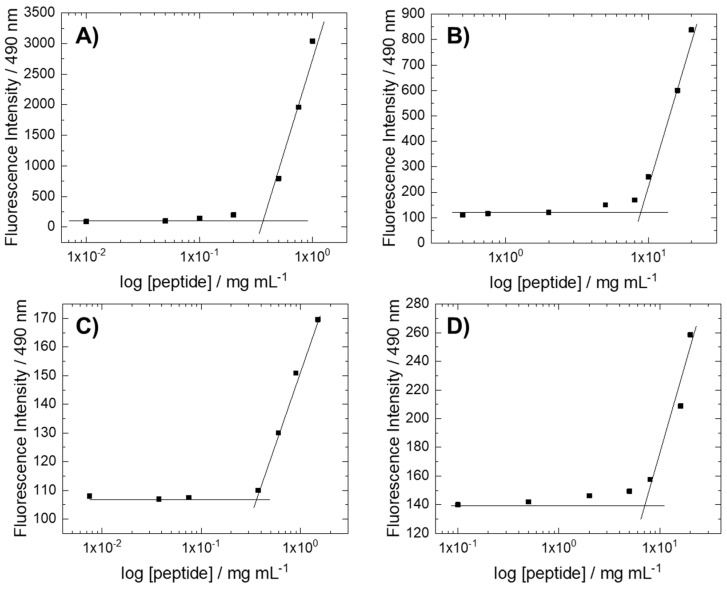
CAC determination in the breakpoint of graphic obtained reporting the fluorescence intensity at 482 nm as a function of the peptide concentration: (**A**) GAIIGL, (**B**) NNQQ, (**C**) GNNQQNG and (**D**) SSTSAA.

The aggregation propensity of these peptides in PBS was further analyzed by measuring the critical aggregation concentration (CAC). Attempts to determine the CAC using 8-anilino-1-naphthalenesulfonic acid (ANS), a fluorophore that typically shows an increase in intensity and a blue shift in its emission upon partitioning into hydrophobic environments, yielded no measurable or interpretable response for any of the four peptides. Accordingly, supported by the clear evidence of prevalent β-type structuration obtained in the preceding experiments, CAC measurements were subsequently performed using ThT, which is specifically suited for β-rich amyloid-like assemblies. The CAC values, determined by monitoring the ThT fluorescence intensity at 485 nm as a function of peptide concentration and calculated at the curve’s break point, were found to be 0.365 mg/mL for GAIIGL, 8.97 mg/mL for NNQQ, 0.363 mg/mL for GNNQQNG, and 7.67 mg/mL for SSTSAA (Figure 8 and Table 1). These values suggest that the aggregation propensities of the peptides are globally anti-correlated with their solubilities. The low solubility and, therefore, the tendency to precipitate shown by GAIIGL and GNNQQNG likely drives their remarkable aggregation propensity. On the other hand, although NNQQ and SSTSAA present remarkable differences in the solubility (10 versus 16 mg/mL), they have similar CAC values (8.97 and 7.67 mg/mL). The relatively higher tendency for SSTSAA to form amyloid-like aggregates compared to NNQQ may be due to the shorter length and smaller size of the side chains of its residues.

#### 2.3.3. Scanning Electron Microscopy

As done for the aggregates formed from DMSO/HEPES solutions, the supramolecular morphology of peptide aggregates grown from PBS was examined by SEM. Samples drop-cast onto aluminium stubs and air-dried showed predominantly compact, densely packed deposits rather than well-resolved individual fibrils across all four sequences, similar to the DMSO/HEPES-prepared samples (Figure S8).

#### 2.3.4. Analysis of the Visible Probe-Free Fluorescence in PBS

The intrinsic label-free fluorescence emission of these peptides in PBS solution was measured at their maximum solubility, as done for the structural characterizations (see above). Surprisingly, in contrast to what was observed in DMSO/HEPES solution, none of the peptides exhibited visible fluorescence emission when excited in the 290–500 nm range (Figure S8E). Among other factors, one possible explanation for this behavior is the limited solubility of these peptides in PBS solution, which is generally only about twice the CAC values, a ratio significantly smaller than that commonly observed. In this concentration regime, the fraction of supramolecular assembled species in bulk solution, prior to precipitation, may be too low to produce a detectable emission signal. Consistent with these considerations, when the same highly soluble solutions were drop-cast onto glass microscope slides, dried, and examined using fluorescence optical microscopy, the resulting deposits displayed detectable visible fluorescence, with the signal under the GFP filter being the most prominent (Figure S9A–D).

## 3. Discussion

The ability of amyloid-like assemblies with a cross-β structure to emit fluorescence across a wide range of the visible spectrum is a puzzling phenomenon with remarkable implications for both basic sciences and applied fields. Interestingly, the emergence of this fluorescence is governed by purely structural effects, since it occurs independently of the presence of a fluorescent side chain in the assembled peptides. Despite remarkable efforts over the last decade, the physicochemical basis of this phenomenon remains elusive, although some intriguing hypotheses have been proposed [32,33,34,35,36]. Mechanistic models investigated over the years consider intermolecular proton transfer occurring along hydrogen bonds connecting N- and C-termini of opposite [33], the occurrence of short hydrogen bonds [35] charge-transfer excitations [51], deplanarization of the amide groups [34], or a carbonyl-lock mechanism [37] in a rigid environment of the cross β-sheet arrangement. Among the reasons for this puzzling situation is the frequent missing link between atomic-level structural studies and spectroscopic investigations, as most studies in the literature focus on only one of these two aspects. In this scenario, we investigated the spectroscopic properties of peptide systems composed exclusively of non-aromatic residues, which played a key role in advancing structural understanding of the principles governing amyloid-like aggregation through crystallographic [15,16] and molecular dynamics simulations [44,45,46,47,48,49,51,52,53,54,55,56,57,58,59,60,61,62,63]. In line with the previous literature indications [15,16,39,40,41,42,43,44,45,46,47,48], three of these peptides (GAIIGL, NNQQ, and SSTSAA) show a strong tendency to aggregate. We also show that the peptide GNNQQNG, a variant of the widely used model system GNNQQNY devoid of aromatic residues, can form amyloid-like aggregates, suggesting the importance of the NNQQ core in the aggregation of these peptides. The characterization of the spectroscopic properties of their self-assembled states unravels an apparently puzzling situation. Indeed, fluorescence emission in solution is observed in DMSO/HEPES, but not in PBS. A careful characterization of peptide solubility in these two media suggests that this may be due to the low degree of aggregation achievable in PBS. However, it cannot be ruled out that the different properties of the two media, especially hydrophobicity, may have a distinct impact on the putative mechanistic models illustrated above. A different scenario emerges in solid-state experiments that show that samples obtained from both media exhibit the phenomenon. For all peptides, the emitted fluorescence spans a rather wide range of wavelengths. Although centered in the blue region, a significant signal is also observed in the green region, independent of their physical state. This property is relatively uncommon among amyloid-like peptides that, in solution, generally emit exclusively in the blue region.

Finally, the structural analysis by 2D GIWAXS of the solid-state properties of the fluorescent material reveals features related to peptide packing in the single crystal. This observation suggests that the three-dimensional crystal structures of these peptides may serve as reliable models for studies aimed at correlating structural, electronic, and spectroscopic features. In this scenario, the present findings represent a valuable benchmark for current and future structural and electronic explanations of the unusual structure-induced amyloid fluorescence.

## 4. Materials and Methods

### 4.1. Chemicals

Protected N^α^-Fmoc-amino acid derivatives, pre-loaded Wang Resins, and peptide coupling reagents were obtained from Calbiochem–Novabiochem (Laufelfingen, Switzerland). Unless otherwise specified, all additional chemicals were sourced from Fluka (Buchs, Switzerland), Merck (Milan, Italy), or Labscan (Stillorgan, Dublin, Ireland) and used as received.

### 4.2. Solid-Phase Peptide Synthesis

The synthesis of the peptide derivatives GAIIGL, NNQQ, GNNQQNG, and SSTSAA (see Figure 1) was carried out using the standard solid-phase peptide synthesis (SPPS) method, employing the Fmoc/tBu protection strategy and Wang-type resins preloaded with the first amino acid of each sequence. The synthesis scale was set at 0.2 mmol for the GAIIGL, GNNQQNG, and SSTSAA derivatives, and at 0.3 mmol for the NNQQ derivative. Prior to synthesis, the resin was swollen in DMF for 30 min. Fmoc deprotection was performed by two consecutive 10-min treatments with 30% (*v*/*v*) piperidine in DMF. A two-fold molar excess of each Fmoc-protected amino acid was coupled using O-(7-azabenzotriazol-1-yl)-*N*,*N*,*N′*,*N′*-tetramethyluronium hexafluorophosphate (HATU) as the coupling reagent in the presence of a four-fold molar excess of *N*,*N*-diisopropylethylamine (DIPEA) in DMF. Each coupling step was performed twice, with a 40-min reaction time for each cycle. Upon completion of synthesis, the resin was washed with diethyl ether and dried. Peptides were cleaved from the resin and fully deprotected using a trifluoroacetic acid (TFA)/triisopropylsilane (TIS)/H_2_O mixture (92.5:5:2.5 *v*/*v*/*v*) for 3 h at room temperature. The crude peptides were precipitated with cold diethyl ether and lyophilized three times.

Purification was carried out by reverse-phase high-performance liquid chromatography (RP-HPLC) using an Agilent 1260 Infinity II Manual Preparative LC System (Agilent, Santa Clara, CA, USA) equipped with a Phenomenex C18 column (Torrance, CA, USA). The mobile phase consisted of solvent A (H_2_O with 0.1% TFA) and solvent B (acetonitrile with 0.1% TFA), with a linear gradient of solvent B from 15% to 70% over 25 min at a flow rate of 20 mL/min. Peptide purity was assessed *via* analytical RP-HPLC on an Agilent 1260 Infinity II LC System, employing the same solvent system and column, with a gradient from 5% to 70% B over 20 min at a flow rate of 1 mL/min. Peptide identity was confirmed by electrospray ionization mass spectrometry (ESI-MS) using an LTQ XL Linear Ion Trap Mass Spectrometer (Thermofisher, Ferentino, Italy) (see Table 1).

### 4.3. Sample Preparation

#### 4.3.1. PBS Samples

Peptide solutions were prepared by dissolving the lyophilized powder in 10 mmol/L PBS (pH = 7.4) at their maximum solubility. Samples were sonicated for 15 min to ensure complete dissolution.

#### 4.3.2. DMSO/HEPES Samples

Lyophilized peptides were first dissolved in dimethyl sulfoxide (DMSO) at 5 mM concentration and then diluted in a buffer containing 150 mM HEPES (pH 7.4) and 150 mM NaCl to reach a final peptide concentration of 0.5 mmol/L. The solutions were then allowed to age for 24 h.

### 4.4. Circular Dichroism (CD) Spectroscopy

Peptide solutions were analyzed using a 0.1-cm-pathlength quartz cuvette. Far-UV circular dichroism (CD) spectra were acquired at 25 °C using a Jasco J-810 spectropolarimeter equipped with a NesLab RTE111 temperature control unit (JASCO Europe, Cremella, Italy). Spectral data were collected over the wavelength range from 280 to 190 nm, using a step size of 0.5 nm, a bandwidth of 1 nm, and an integration time of 1 s per step. For each sample, three individual scans were recorded, averaged, and baseline-corrected by subtracting the appropriate blank spectrum. The resulting spectra, originally in optical density, were then converted to mean residue ellipticity (Θ, expressed in deg·cm^2^·dmol^−1^) using the following equation:

[Θ] = millidegrees/(pathlength in millimeters × concentration in M × number of peptide bonds)

### 4.5. Fourier Transform Infrared (FTIR) Spectroscopy

FTIR spectra for dried samples were collected using a Jasco FT/IR 4100 spectrometer (Easton MD). Peptide solutions were placed in a Specac Pearl liquid cell with CaF_2_ plates. For data acquisition, liquid solutions were left to dry on the cells before the experiment was carried out. Each sample underwent a total of 128 scans. Spectra were collected over the wavenumber range 900–4000 cm^−1^ (sensitivity of 1.0 cm^−1^).

### 4.6. Fluorescence Spectroscopy

Fluorescence measurements for solution samples were performed using a Shimadzu RF-6000 (Kyoto, Japan) spectrofluorometer. Samples were analyzed in a quartz cuvette measuring 10.0 mm × 5.00 mm. Instrumental settings included excitation and emission bandwidths of 2.5 nm, and all measurements were conducted at a controlled temperature of 25 °C. Thioflavin T (ThT) was employed to determine the critical aggregation concentration (CAC) of the peptide analogues [49]. Peptide solutions of varying concentrations were prepared with ThT at a fixed concentration of 5 × 10^−5^ mol/L. Following excitation at 450 nm, emission spectra were recorded in the range of 460–600 nm. The CAC was determined by plotting fluorescence intensity at 490 nm against peptide concentration. Additionally, emission spectra of the peptides in the absence of ThT were acquired following excitation at different wavelengths; finally, spectra were also recorded in excitation mode. Quantum yield measurements were performed using a Jasco FP8550 spectrofluorometer equipped with an integrating sphere. Samples were prepared by drop-casting peptide solutions onto glass slides, which were then left to dry overnight under ambient conditions to ensure complete solvent evaporation. All measurements were performed at 25 °C, with baseline corrections using a blank glass slide as the reference.

### 4.7. Fluorescence Microscopy

Peptide solutions were drop-cast onto microscope slides and left to dry overnight. The resulting solid-state peptide aggregates were analyzed using a Fluorescence Optech MB80-Pol microscope (San Prospero, Italy) equipped with 4′,6-diamidino-2-phenylindole (DAPI; λ_ex_ = 400–410 nm, λ_em_ = 455 nm) and green fluorescent protein (GFP; λ_ex_ = 460–495 nm, λ_em_ = 505 nm) filters.

### 4.8. Scanning Electron Microscopy (SEM)

Morphological analysis of the aggregates was performed using field-emission scanning electron microscopy (FE-SEM) on a Phenom ProX instrument (Thermo Fisher Scientific, Waltham, MA, USA). Samples were prepared by drop-casting 10 μL of peptide solution onto an aluminium stub and air-drying. A 7 nm gold coating was applied by sputtering with a LUXORau SEM coater (Aptco Technologies, Nazareth, Belgium) at 25 mA current for 75 s. The sputter-coated samples were then transferred to the specimen chamber, and images were acquired at an accelerating voltage of 15 kV using the Secondary Electron Detector (SED) (RIT (Rigaku Innovatibe Technologies), Auburn Hills, MI, USA).

### 4.9. Grazing Incidence Wide-Angle X-Ray Scattering (GIWAXS)

GIWAXS measurements were performed at the XMI-Lab [64], equipped with a Fr-E+ Superbright X-ray micro-source (Rigaku, Tokyo, Japan) coupled to an SMAX3000 SAXS/WAXS camera (RIT (Rigaku Innovatibe Technologies)) via a focusing optics and a three-pinhole beam shaping system delivering a 0.2 mm (diameter) beam spot at the sample position. A Fuji Image Plate detector was employed, placed downstream of the sample at a distance of 200 mm, and read offline by a Raxia scanner (RIT (Rigaku Innovatibe Technologies)). GIWAXS data were processed by using the in-house developed program SUNBIM [65]. GIWAXS data were calibrated by using Ag Behenate powder standard, and a flat detector correction was applied as implemented in SUNBIM, without any baseline interpolation. A high-precision goniometer with piezoelectric motors was used for sample alignment: the incidence angle was set to 0.18° or 1° for all measurements to maximize diffraction intensity. Samples were kept at about 0.1 mbar vacuum pressure during measurements.

## Figures and Tables

**Table 1:** Formula, theoretical and experimentally found molecular weight (MW), logP and CAC values, expressed in mmol/L and in mg/mL, for non-aromatic peptides. CAC values have been determined in the break point of the plots in Figure 8.

Peptide	Formula	MW_calc._ (a.m.u.)	MW_deter._ (a.m.u.)	LogP	CAC (mg/mL)	CAC (mmol/L)
GAIIGL	C_25_H_46_N_6_O_7_	542.7	543.3	1.73 ± 0.80	0.365	0.673
NNQQ	C_18_H_30_N_8_O_9_	502.5	503.2	−4.92 ± 0.87	8.97	17.9
GNNQQNG	C_26_H_42_N_12_O_13_	730.7	731.4	−7.13 ± 0.94	0.363	0.497
SSTSSA	C_19_H_34_N_6_O_11_	522.5	523.1	−3.84 ± 0.86	7.67	14.7

## Data Availability

The original contributions presented in this study are included in the article/Supplementary Materials. Further inquiries can be directed to the corresponding authors.

## Supplementary Materials

The following supporting information can be downloaded at https://www.mdpi.com/article/10.3390/ijms27146453/s1.
