# Supplementary material for: The Intrinsic UV–Visible Fluorescence of Peptides Widely Used for Studying Amyloid Aggregation Devoid of Aromatic Residues
Source: Int J Mol Sci. 2026 Jul 20;27(14):6453. doi: 10.3390/ijms27146453 (PMC13410050; doi:10.3390/ijms27146453)
Supplement: Supplementary file 1 [file ijms-27-06453-s001.zip › ijms-4361606-supplementary.pdf]

Supplementary Information

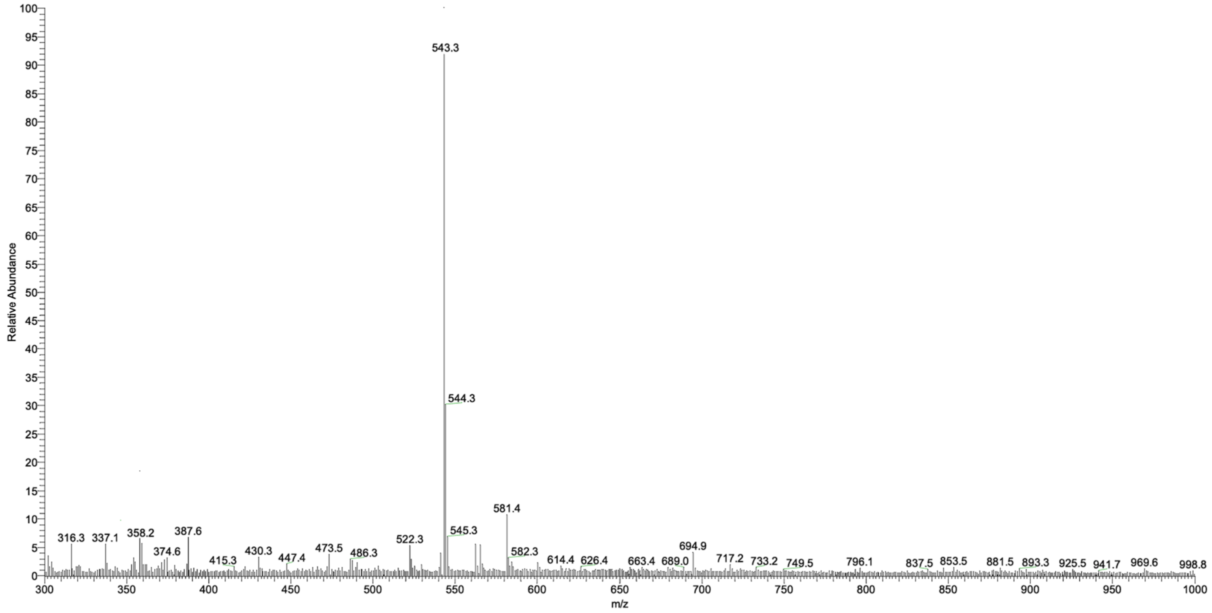

Figure S1: ESI-MS spectrum of GAIIGL peptide

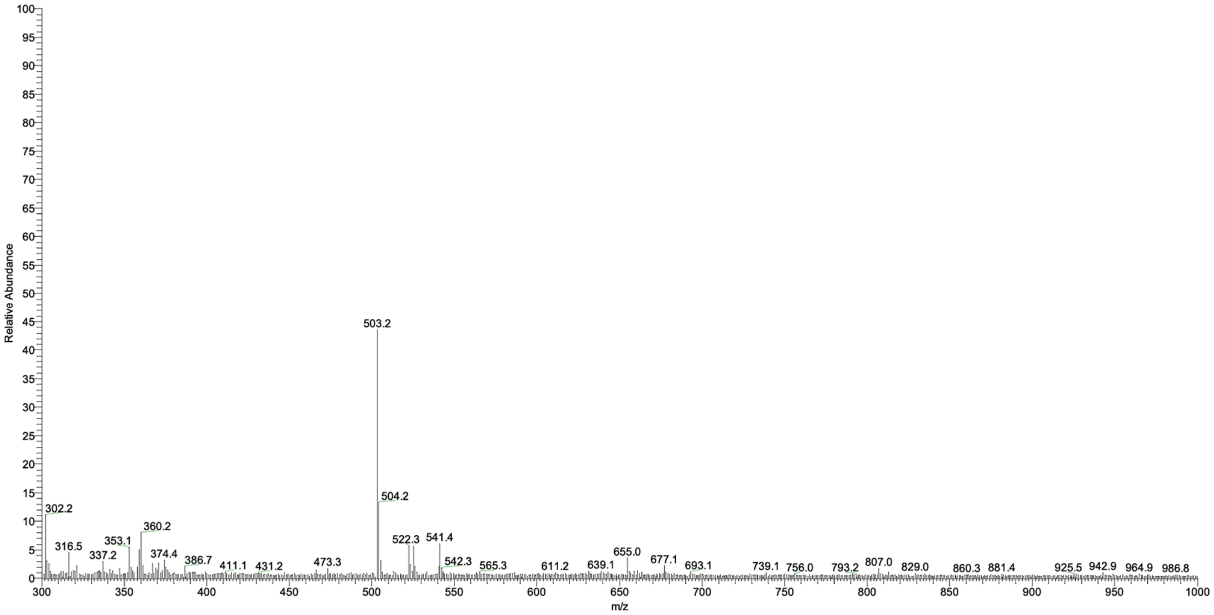

Figure S2: ESI-MS spectrum of NNQQ peptide

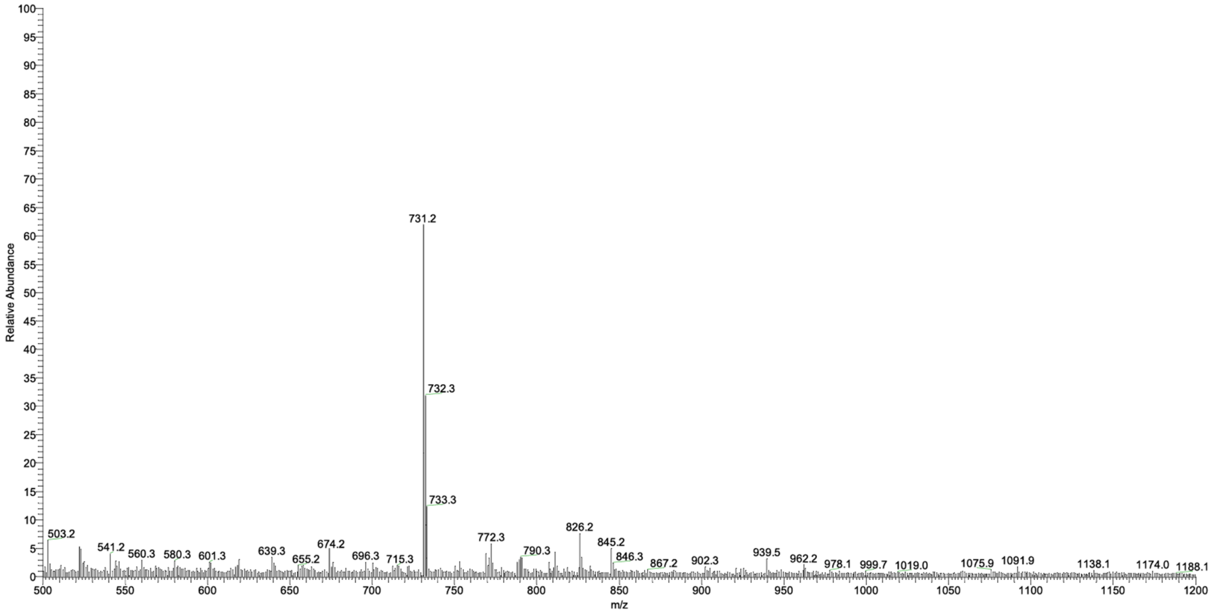

Figure S3: ESI-MS spectrum of GNNQQNG peptide

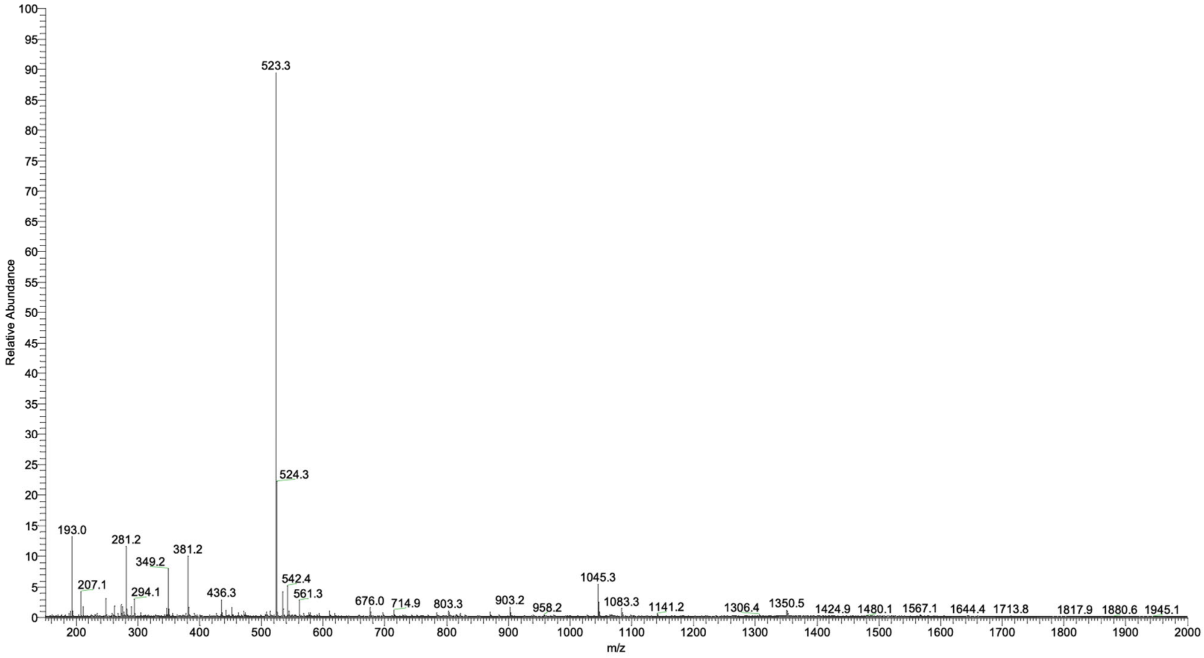

Figure S4: ESI-MS spectrum of SSTSAA peptide

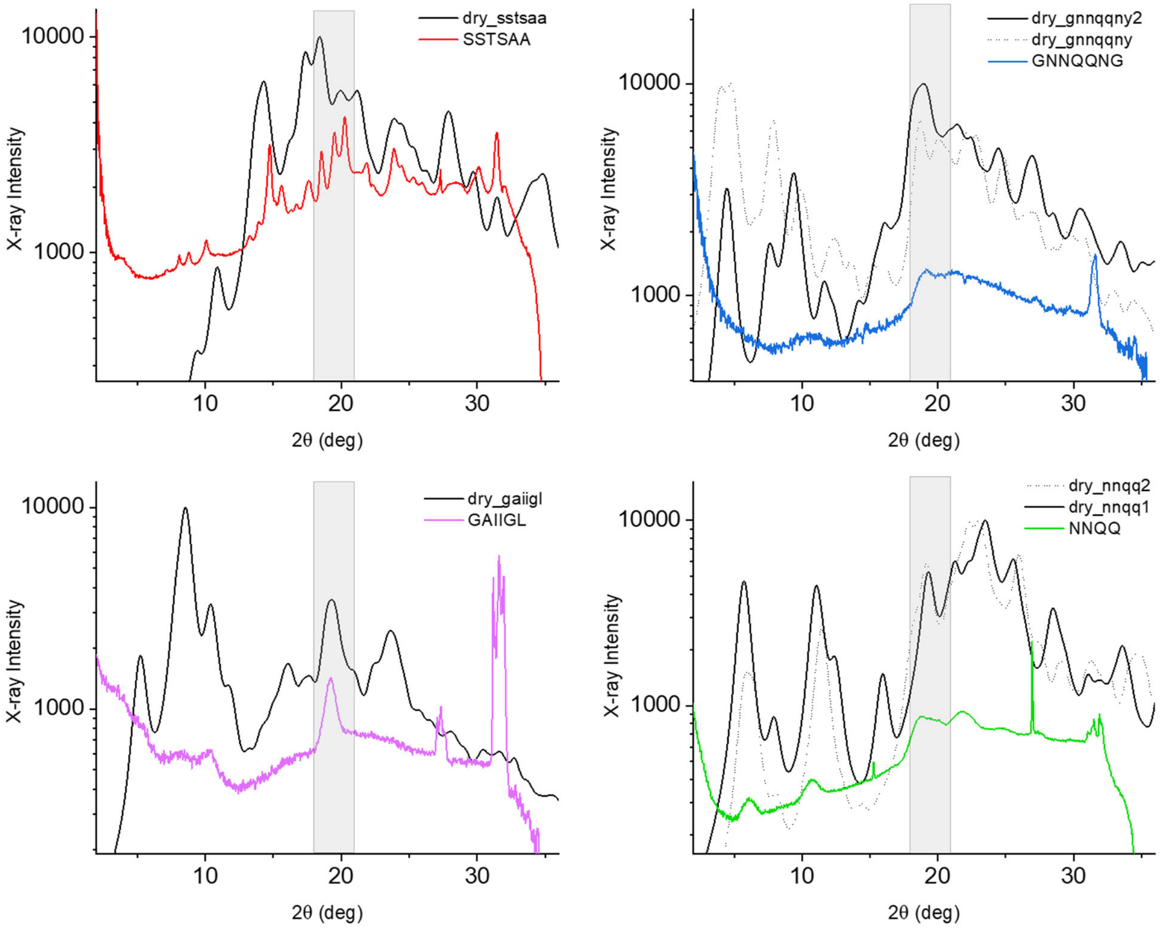

Figure S5: Experimental (1D-folded GIWAXS) and calculated (from PDB, https://people.mbi.ucla.edu/sawaya/jmol/xtalpept/index.html) diffraction patterns for the selected peptide sequences: SSTSAA and GAIIGL (one dry form); GNNQQNG and NNQQ (two dry forms).

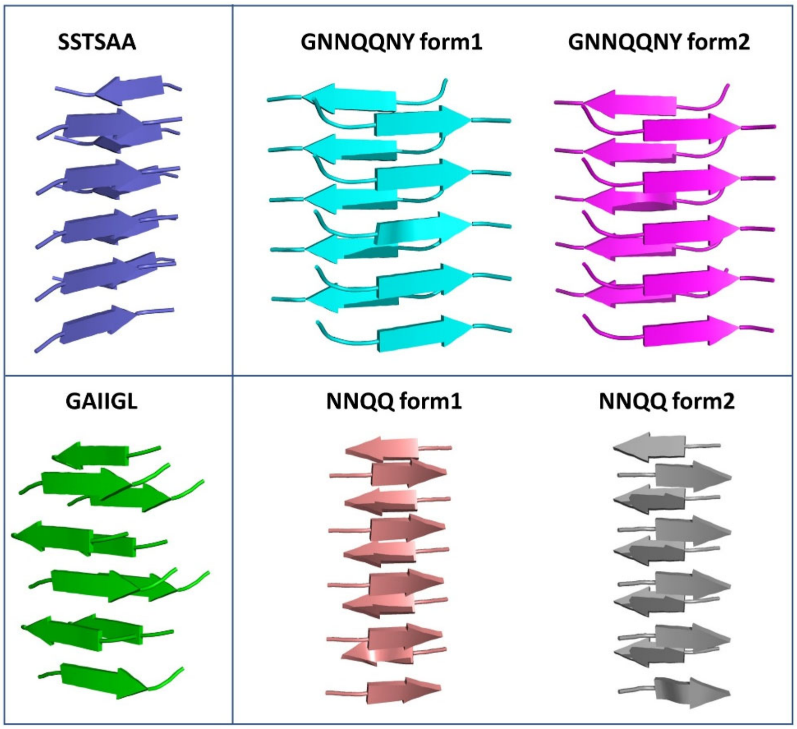

Figure S6: Reference models (generated from https://people.mbi.ucla.edu/sawaya/jmol/xtalpept/index.html) for pairs of β-sheets structures of the selected peptide sequences: SSTSAA and GAIIGL (one dry form); GNNQQNG and NNQQ (two dry forms).

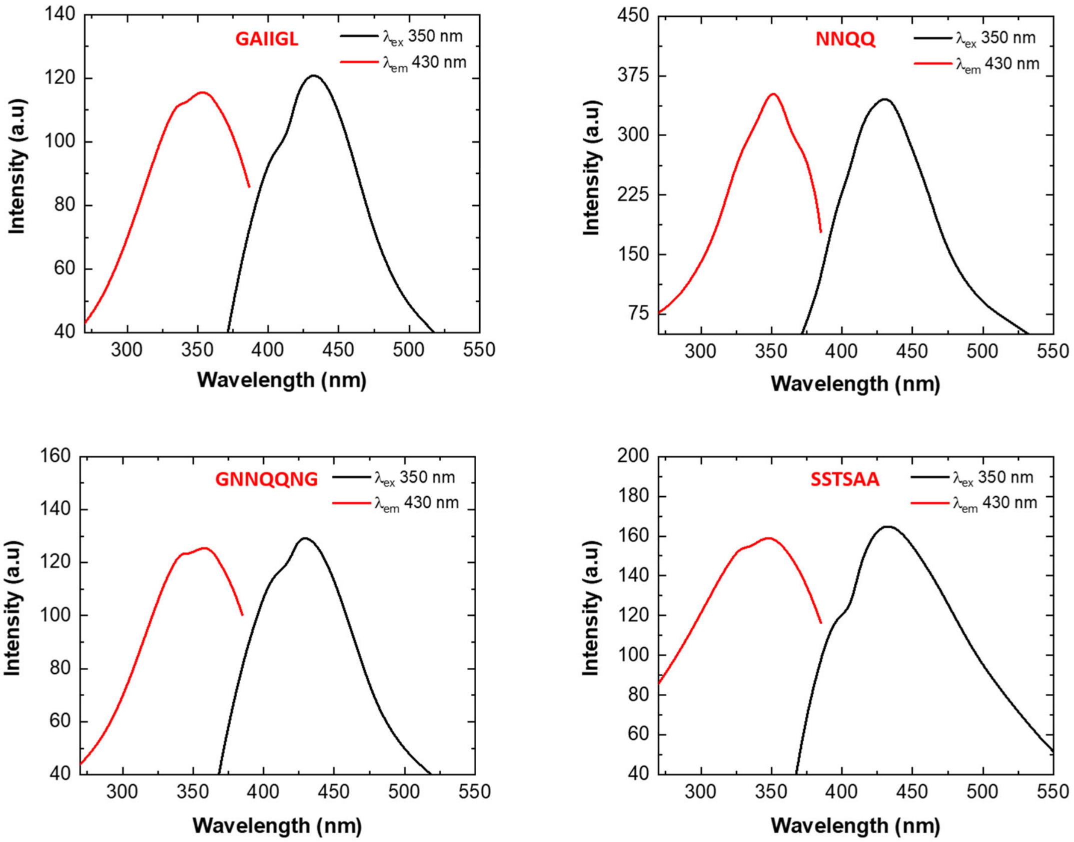

Figure S7: Fluorescence emission and excitation spectrum for each peptide dissolved in DMSO/HEPES.

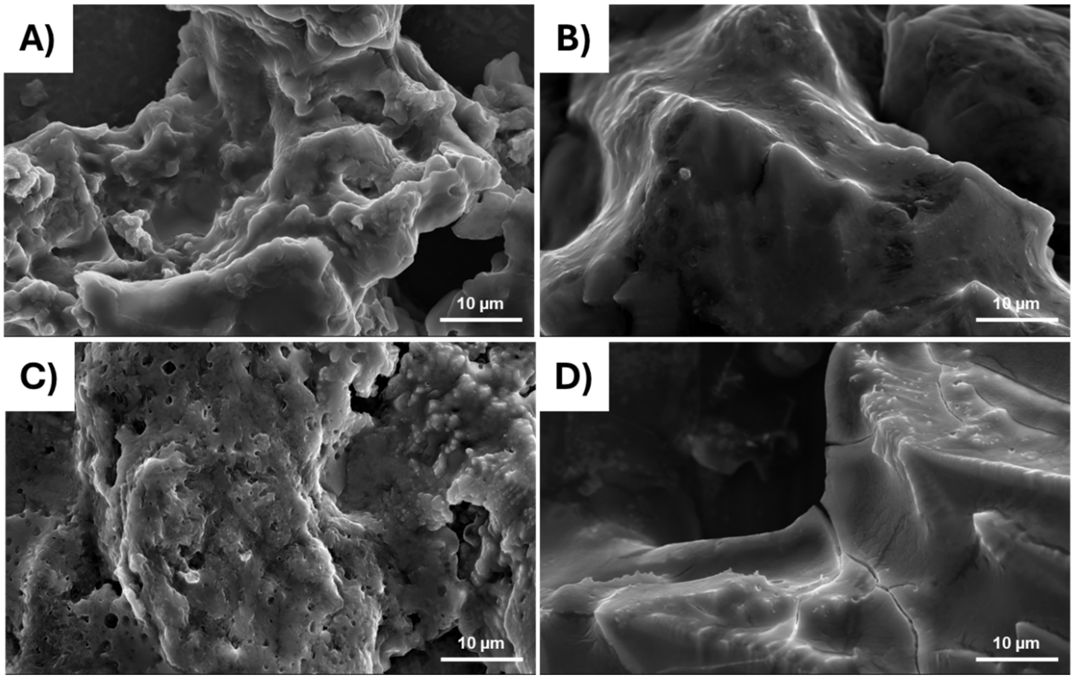

Figure S8: SEM micrographs of supramolecular aggregates formed by the four peptide sequences under the PBS preparation protocol: **(A)** GAIIGL, **(B)** NNQQ, **(C)** GNNQQNG, and **(D)** SSTSAA (scale bars as indicated).

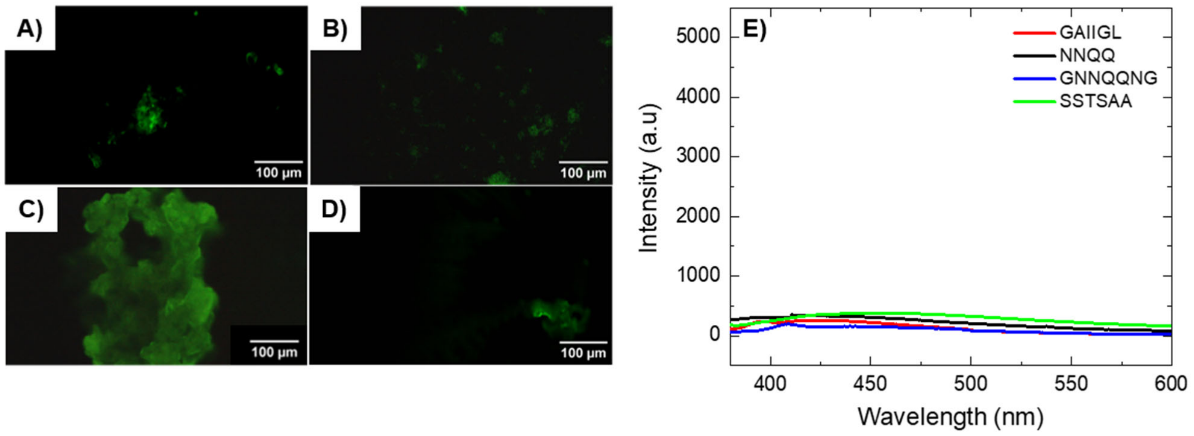

Figure S9: Fluorescence microscopy images of GAIIGL **(A)**, NNQQ **(B),** GNNQQNG **(C),** and SSTSAA **(D)** peptide solutions drop-casted on glass. Spectroscopic characterization of peptide solutions at their maximum concentration, excitation wavelength 350 nm **(E)**.

Table S1: SAXS data for the non-aromatic peptides.

GAIIGL			GNNQQNG			NNQQ			SSTSAA		
2θ	q (1/nm)	d(nm)	2θ	q (1/nm)	d(nm)	2θ	q (1/nm)	d(nm)	2θ	q (1/nm)	d(nm)
3.95	2.81	2.24									
5.13	3.66	1.72									
						6.13	4.36	1.44			
8.02	5.70	1.10							8.09	5.75	1.09
									8.79	6.25	1.00
10.28	7.31	0.86							10.05	7.15	0.88
			10.68	7.59	0.83	10.82	7.69	1.63			
									13.24	9.40	0.67
									10.70	10.44	0.60
			14.43	10.25	0.61						
						15.18	10.77	0.58			
									17.62	12.49	0.50
						18.75	13.29	0.47	18.54	13.14	0.48
19.32	13.69	0.46	19.185	13.60	0.46				19.50	13.81	0.45
						20.33	14.40	0.44	20.26	14.35	0.44
						21.66	15.33	0.41	21.86	15.47	0.41
						24.73	17.46	0.36			
27.11	19.12	0.33	27.10	19.11	0.33	26.96	19.02	0.33			
27.95	19.70	0.32									
31.59	22.20	0.28	31.51	22.15	0.28				31.67	22.26	0.28
						33.21	23.31	0.27			
